# Mechanistic insights of substrate transport and inhibitor binding revealed by high-resolution structures of human norepinephrine transporter

**DOI:** 10.1038/s41422-024-01024-0

**Published:** 2024-09-02

**Authors:** Ailong Song, Xudong Wu

**Affiliations:** 1 Key Laboratory of Structural Biology of Zhejiang Province, School of Life Sciences, Westlake University, Hangzhou, Zhejiang China; 2 Westlake Laboratory of Life Sciences and Biomedicine, Hangzhou, Zhejiang China; 3 Institute of Biology, Westlake Institute for Advanced Study, Hangzhou, Zhejiang China

**Keywords:** Cryoelectron microscopy, Transport carrier

Dear Editor,

Norepinephrine (NE), also known as noradrenaline, is an important neurotransmitter that plays crucial roles in the central nervous system, including the regulation of mood, memory, and alertness. It also functions as a key hormone in the whole body. In the brain, NE transporter (NET, encoded by the *SLC6A2* gene), a Na^+^/Cl^–^-dependent transporter, reuptakes released NE at neuron synapses, thus maintaining NE homeostasis. Due to the important functions of NET, many drugs targeting NET have been developed to treat various neurological disorders.^1^ Despite previous efforts using NET surrogate to gain insights into the transport mechanism,^2^ the specific recognition of NE and the mechanistic details of the transport process remain elusive. Moreover, the binding modes of several FDA-approved NET-selective inhibitors require further investigations to provide a solid model for future structure-based drug discovery.

In this study, we report high-resolution cryo-EM structures of human NET (hNET) bound with different ligands in three distinct states: an NE-bound inward-open state, an NE-bound occluded state, and an outward-open state bound with inhibitors. These structures reveal intricate details of NE recognition and inhibitor binding, and highlight the significant involvement of water molecules in such recognition process. The comparison of conformations of three different states provides mechanistic insights into the transport process mediated by hNET.

We first attached a FLAG-avi tag to the N-terminus of full-length hNET and an N-terminal truncated version, hNET (47–617), and then purified the proteins in the presence of different ligands. Some populations of purified proteins were found to be highly glycosylated (Supplementary information, Fig. S1a, b). The yield and purity of FLAG-avi-hNET (47–617) (denoted as hNETcryo) were better and hNETcryo exhibited similar transport activity for NE and displayed similar sensitivities to different inhibitors compared to wild-type (WT) hNET (Supplementary information, Figs. S2a, c and S3). Therefore, hNETcryo purified in detergent was used for further cryo-EM analysis. To facilitate cryo-EM studies, we performed nanobody screenings (details are provided in Supplementary information) and chose two hits after extensive validations. Nb_BF9, screened using NE-bound hNETcryo, did not interact with inhibitor-bound hNET (data not shown). Therefore, Nb_BF9 was only used to form a complex with hNETcryo_NE. Another hit Nb_BB4, obtained using reboxetine-bound hNET, can form complexes with hNETcryo_NE, hNETcryo_reboxetine, and hNETcryo_atomoxetine (Supplementary information, Fig. S1c). These two nanobodies were able to attenuate the transport activity of NE (Supplementary information, Fig. S2b) while displaying selective binding properties, suggesting that they are conformation-specific binders.

We then subjected these four complexes to cryo-EM analysis and determined their high-resolution structures at overall resolutions of 2.4–2.6 Å assessed by the gold standard of FSC  =  0.143 (Fig. 1a; Supplementary information, Figs. S4, S5 and Table S1). Each structure consists of a monomer of hNETcryo in complex with one nanobody. The high-quality maps facilitated the identifications of ligands/ions/water molecules/lipids, the accurate assignment of side-chain rotamers, and the carbonyl oxygen of peptide bonds (Supplementary information, Figs. S6, S7). These details turned out to be crucial for a comprehensive understanding of the recognition of NE and inhibitors.

**Fig. 1:**
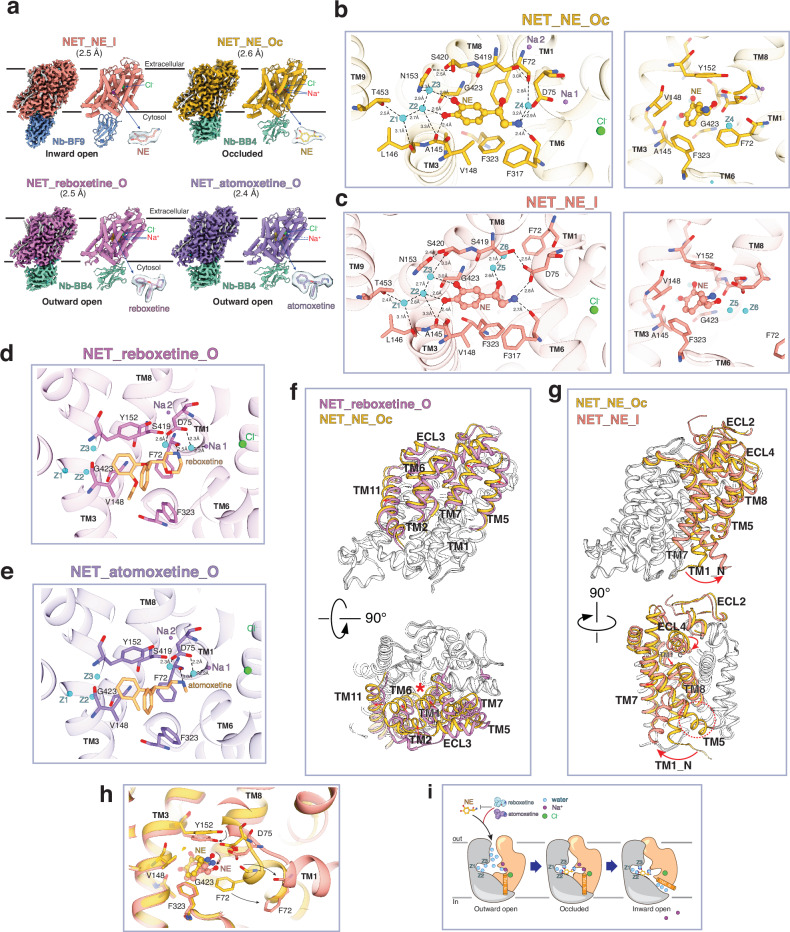
Mechanistic insights into the NE recognition, transport, and inhibitor binding of hNET. **a** The cryo-EM density maps and built models are shown. The ligands are highlighted and shown as sticks with local density maps in semi-transparent blue. Cl^–^ and Na^+^ ions are also indicated. **b**, **c** Different views of the substrate-binding site in NET_NE_Oc (**b**) and NET_NE_I (**c**), with NE and nearby residues shown as sticks. The peptide backbones are shown in the cartoon. Water molecules, denoted as Z, are shown in cyan. For simplicity, only water molecules that are in close contact are shown. The dashed lines with numbers indicate the distances between different atoms. **d**, **e** Inhibitor-binding sites are shown for NET_reboxetine_O (**d**) and NET_atomoxetine_O (**e**), similar to panels **b** and **c**. **f** Models of NET_reboxetine_O and NET_NE_Oc are aligned and shown in cartoon in different views. The parts in light gray indicate regions with very minimal movement. Transmembrane segments and ECLs between the transmembrane segments, which exhibit larger movements, are color-coded. The red arrows indicate the directions of the movements. The red asterisk indicates the NE-binding site. **g** Similar to **f** but for the alignments of NET_NE_Oc and NET_NE_I. The red dashed circle indicates the absence of TM5’s helical structure in NET_NE_I. **h** Close-up view of panel (**g**) on the substrate-binding site. **i** Schematic model for the recognition and transport of NE by hNET.

Based on the obtained structures, hNETcryo_reboxetine and hNETcryo_atomoxetine exhibited an outward-open conformation, featured by an open extracellular cavity. These structures are labeled as NET_reboxetine_O and NET_atomoxetine_O, respectively. The two structures are almost identical (RMSD of 0.18 Å) (Supplementary information, Fig. S9a). hNETcryo_NE in complex with Nb_BB4 adopted an occluded conformation where both the extracellular and cytosolic cavities are closed, and this structure is referred to as NET_NE_Oc. In the complex of hNETcryo_NE with Nb_BF9, an inward-open conformation with the cytosolic cavity open was observed, thus denoted as NET_NE_I. NET_reboxetine_O, previously determined reboxetine-bound *Drosophila* dopamine transporter (dDAT) (dDAT_reboxetine_O) and inhibitor-bound human serotonin transporter (hSERT) (hSERT_paroxetine_O) share a high degree of overall structural similarity, with notable differences in the extracellular loop (ECL) between TM3 and TM4 (ECL2) and the very C-terminus (Supplementary information, Fig. S9b). The resolved parts of ECL2 in hNET show that it folds against the ECL between TM7 and TM8 (ECL4). This tight association generates a more compact “lid” occluding the extracellular vestibule in the inward-facing state. Unlike dDAT and hSERT structures, the complete C-terminus of hNET (Q581–I617) was well resolved. This region forms a “latch” consisting of several short alpha helices that interact with the intracellular loop (ICL) between TM2 and TM3 (ICL1), as well as the cytosolic ends of TM10 and TM11.

Ligands in all structures can be accurately modeled (Fig. 1a). Based on previous studies on other SLC6 family members and our cryo-EM densities, Na^+^ and Cl^–^ ions were assigned. In NET_reboxetine_O, NET_atomoxetine_O, and NET_NE_Oc, one Cl^–^ and two Na^+^ were identified. The density for Na 1 in NET_atomoxetine_O is relatively weaker, indicating higher flexibility or lower occupancy. In NET_NE_I, one Cl^–^ but no Na^+^ was identified (Supplementary information, Fig. S8a). Despite potential uncertainties, approximately 50–80 water molecules were modeled in different structures based on strong densities connected to protein components or other water molecules, as well as their chemical environment. In all structures, a lipid molecule was identified at the same location, with side chains of K135, Y139, Y572, and Y575 coordinating the phosphate group. Considering the size of density for the lipid headgroup and potential clashes with nearby residues, this lipid is currently modeled as Phosphatidylethanolamine, but the lipid-raft enriched sphingolipid could be an alternative fit (Supplementary information, Figs. S6, S7, S8c). An allosteric site for serotonin has been identified in hSERT.^3^ Only in NET_NE_I, we observed an elongated density that resembles a second molecule of NE (NE-2) in an exposed shallow pocket near ECL4 and is coordinated by E382, R301, and several hydrophobic residues (Supplementary information, Fig. S6a). However, given the weak density, high concentration of NE (2 mM) used during purification, and relatively low physiological concentration of NE (typically in the µM range), NE-2 was not modeled.

The high-resolution structures of NET_NE_Oc and NET_NE_I reveal comprehensive details of NE recognition. NE was identified within a central cavity in the middle of transmembrane region, which can be further divided into three Subsites: A, B and C.^4^ Residues in TM1/6/8 sculpt Subsite A (SA), while residues in TM3/TM8 shape Subsite B (SB) and residues in TM3/6/10 form Subsite C (SC). The interactions of NE directly involve TM1, TM3, TM6 and TM8, and indirectly involve TM9. In NET_NE_Oc, NE binding is stabilized by two types of interactions. The benzene ring of the catechol moiety of NE is stabilized in a pocket formed by side chains of F72 (SA), A145/Y152/G423 (SB), and V148/ /F323 (SC) mainly through hydrophobic interactions (Fig. 1b, right panel). Interestingly, the polar interactions are not primarily mediated by side chain interactions but by the carbonyl oxygens of the peptide bond and water molecules. Specifically, the two hydroxy groups of the catechol moiety form hydrogen bonds (H-bonds) with the carbonyl oxygen of A145 and two water molecules (Z2 and Z3). The amine in the ethylamine side chain of NE forms H-bonds with the carbonyl oxygen of F317 (SC) directly and of F72 indirectly through a water molecule (Z4), as well as with the side chain of D75 (SC) (Fig. 1b, left panel). The water triad (Z1, Z2, and Z3) within SB is stabilized through an intricate H-bond network formed by the carbonyl oxygens of A145/L146/S420 (SB), and side chains of N153 (SB) and T453 on TM9. In NET_NE_I, the overall interaction modes are similar to those in NET_NE_Oc, except that the interaction is weakened due to the displacement of F72 (Fig. 1c). The benzene ring of NE accommodated in the hydrophobic pocket becomes exposed to the solvent. As a result, the carbonyl oxygen of F72 can no longer contribute to the stabilization of the ethylamine side chain of NE. Additionally, the hydroxyl group in the ethylamine side chain of NE is hydrated, interacting with Z5 and Z6.

In human DAT, the residues surrounding the water triad are identical to those in human NET, implying that human DAT may also use the water triad to engage the two hydroxyl groups of dopamine. Indeed, the recently reported dopamine-bound human DAT in an occluded conformation (hDAT_DA_Oc)^5^ shows three water molecules similarly coordinating DA (Supplementary information, Fig. S9c), suggesting a conserved role of water molecules in recognizing NE and DA. While DA is bound in a relatively similar pose in the structure of dopamine-bound dDAT in outward-open conformation (dDAT_DA_O)^6^ (Supplementary information, Fig. S9e), the side chain of D121 (SB) in dDAT occupies a position similar to Z2 and Z3 in hNET, interacting with the two hydroxyl groups of dopamine. This suggests that *Drosophila* has evolved a different mechanism.

The high-resolution structures of NET_reboxetine_O and NET_atomoxetine_O offer a solid model for understanding inhibitor binding (Fig. 1d, e). Both reboxetine and atomoxetine are NET-selective inhibitors. They are observed in the same binding pocket as NE, mainly through hydrophobic interactions with F72 (SA), Y152/G423 (SB), and V148/F323 (SC). In this outward-open state, Y152 (SB) forms an H-bond with the side chain of D75 (SA) that also coordinates two water molecules and Na 1. The secondary amine group of atomoxetine and the morpholine nitrogen of reboxetine form an H-bond with the carbonyl oxygen of F72. It is worth noting that reboxetine exists in two stereoisomers, with the (S,S)-reboxetine being more potent than the (R,R)-isomer.^7^ In our study, reboxetine, specifically as the (S,S)-isomer, fits well into the cryo-EM density. Fitting of an (R,R)-reboxetine would result in slight clashes with the side chain of F72 (Supplementary information, Fig. S9f), assuming that the conformation remains unchanged. This contrasts with a previously reported dDAT_reboxetine_O structure, where the isomer types are ambiguous.^8^ In both NET_reboxetine_O and NET_atomoxetine_O structures, the water triad is observed in the same position as in NET_NE structures (Supplementary information, Fig. S8b, top panel), indicating their pre-existence. The presence of the water triad also raises an intriguing possibility that 4-hydroxy atomoxetine, the major metabolite of atomoxetine, might be even more potent.

Comparing NET_reboxetine_O with NE-bound dDAT in an outward-open conformation (dDAT_NE_O)^2^ reveals a strong structural similarity between the two (RMSD of 0.85 Å) (Supplementary information, Fig. S9d). Therefore, NET_reboxetine_O was used as a surrogate for the currently unavailable NET_NE_O. The structural alignments of hNET in three different states provide insights into the conformational transitions involved in substrate recognition and release. When comparing NET_reboxetine_O and NET_NE_Oc (Fig. 1f), we observed that the transition from the outward-open to occluded state primarily involves the movement of parts of TMs (TM1, 2, 5, 6, 7, and 11) on the extracellular side. The most significant movement is the tilting of TM1, 2 and 6 towards the NE-binding site, which results in the closure of the extracellular cavity. This transition creates additional interactions stabilizing the occluded conformation and sealing the extracellular cavity. For instance, D473 on TM10 interacts with R81 on TM1 (Supplementary information, Fig. S10a), while R81 interacts with E382 in ECL4 in the outward-open state (Supplementary information, Fig. S10b). This finding is consistent with a previous study showing that mutating D473 to alanine abolishes NE transport.^9^

Based on the alignment of NET_NE_Oc and NET_NE_I (Fig. 1g), three notable movements are observed during the transition from occluded to inward-open state: movement of ECL4 towards the cytosol with accompanying rearrangement of ECL2, the dramatic outward movement of the N-terminal half of TM1 (TM1_N) towards the lipid environment, and the unwinding of helical structures of TM5 that becomes more flexible on the cytosolic side. The reorganization of ECL2 and ECL4 may push the C-terminal half of TM1 (TM1_C) towards the cytosol, leading to the destabilization and dramatic movement of TM1_N. The important roles of ECL2 and ECL4 are consistent with studies showing that mutations of ECL2 and ECL4 residues affect NE transport.^9,10^ In a close-up view (Fig. 1h), F72 from TM1 is displaced from the binding pocket, and Y152/D75/NE tilt towards the cytosol. Through these movements, two bound Na^+^ ions in the occluded state are released, and the cytosolic release cavity becomes open and filled with water molecules, preparing for NE release.

In summary, the high-resolution structures of hNET in three distinct states offer valuable insights into the transport mechanism of hNET (Fig. 1i) and provide a detailed understanding of NE recognition. These structures highlight the significant involvement of multiple water molecules in the central binding cavity for NE recognition. The role of water molecules might go beyond substrate recognition, as water molecules with consistent positions inside the protein across all four structures (Supplementary information, Fig. S8b, bottom panel) are within regions that undergo minimal movement during conformational changes. Furthermore, our study establishes a robust framework for future drug discovery efforts, by accurately resolving the binding mode of reboxetine and atomoxetine, including stereoisomerism of the ligand.

The findings of our study have significant implications for future virtual drug screenings targeting hNET or other SLC6 family members using docking software or AI-based approaches: the role of water molecules needs to be taken into serious consideration. An additional value of the study is the development of two conformation-specific nanobodies that could serve as valuable tools for future drug screening toward specific conformations of hNET and for further structural characterization.

Monomeric and dimeric structures of hNET bound with NE and atomoxetine have been recently reported.^11–14^ The exact source of monomer/dimer difference is unclear, but a previous report indicated that hNET exists in both monomer (60%) and dimer (40%) forms on the membrane.^15^ Regardless, there is little question that the monomer is the functional unit. All atomoxetine-bound hNET structures are very similar. However, there are some differences in the details like the binding pose of atomoxetine (refer to Supplementary information, Fig. S11a, b for details). Monomeric structures of hNET bound with NE in an inward-open state share high similarity (Supplementary information, Fig. S11f), and also resemble the structure of dimeric hNET bound with NE (NET_NE_D) (Supplementary information, Fig. S11c–e). In a more detailed comparison, NE was modeled similarly across all four structures, albeit with different numbers of resolved water molecules (Supplementary information, Fig. S11g).

## Supplementary information

Supplementary Information

## Data Availability

Cryo-EM density maps and atomic coordinates have been deposited in the Electron Microscopy Data Bank and Protein Data Bank under accession numbers EMD-60322 and PDB: 8ZOY, for NET_NE_I; EMD-60331 and PDB: 8ZPB, for NET_NE_Oc; EMD-60324 and PDB: 8ZP1, for NET_reboxetine_O; EMD-60325 and PDB: 8ZP2, for NET_atomoxetine_O.
