# Supplementary material for: Mechanistic insights of substrate transport and inhibitor binding revealed by high-resolution structures of human norepinephrine transporter
Source: Cell Res. 2024 Sep 2;34(11):810–3. doi: 10.1038/s41422-024-01024-0 (PMC11528112; doi:10.1038/s41422-024-01024-0)
Supplement: Supplementary file 1 — Supplementary Information [file 41422_2024_1024_MOESM1_ESM.pdf]

Supplementary Information for

Mechanistic Insights of Substrate Transport and Inhibitor Binding Revealed by High-resolution Structures of Human Norepinephrine Transporter

Ailong Song[1,2,3], Xudong Wu[1,2,3]*

[1]Key Laboratory of Structural Biology of Zhejiang Province, School of Life Sciences, Westlake University, Hangzhou, Zhejiang, China

[2]Westlake Laboratory of Life Sciences and Biomedicine, Hangzhou, Zhejiang, China

[3]Institute of Biology, Westlake Institute for Advanced Study, Hangzhou, Zhejiang, China

*Correspondence to: Xudong Wu (X.W.) (wuxudong@westlake.edu.cn)

This PDF file includes:

Materials and Methods

Figures S1-S11

Tables S1

Reference for methods

Materials and Methods

Expression and purification of NET

The codon-optimized full-length wildtype human NET (Uniprot: P23975) and N-terminal truncated NET (47-617), with an N-terminal FLAG tag and Avi tag including GS linkers, were cloned into a pEG-BacMam vector [1]. The correct plasmids were transformed into DH10Bac for bacmid production. Validated bacmids (10~20ug) were transfected into 1.5×10^6 of Sf9 cells using Cellfectin™ II Reagent (Thermo Fisher Scientific). The cells were incubated at 27°C for 4-5 days for P1 virus production. P1 virus was then used to infect more Sf9 cells for P2 virus production. Subsequently, 33 mL of P2 virus was added to 1 L of Expi293F cells maintained at a density of $2.8\text{-}3 \times 10^6$/mL at 37°C in SMM293-TII expression medium (SinoBiological) supplemented with 1% fetal bovine serum (FBS, cellmax). After 15-16 hours of transduction, sodium butyrate was added to a final concentration of 10mM to enhance protein expression. The cells were cultured for an additional 48 hours at 37°C before being harvested by centrifugation (2,500 g, 15 mins).

The cells were sonicated at 50% strength (2 seconds on /3 seconds off) for a total of 10 mins in a buffer containing 25 mM HEPES pH 7.4, 300 mM NaCl, and 0.5 mM PMSF. After Cell debris was removed by centrifugation at 8,000g for 15 minutes, the supernatant was subjected to ultracentrifugation (43,000 rpm for 1.5 hours at 4°C) to pellet the membrane fractions. The membrane pellet was then resuspended and homogenized using a douncer in buffer (25 mM HEPES pH 7.4, 300 mM NaCl supplemented with protease inhibitor cocktail). Different ligands were added to the membrane suspension at indicated final concentrations (2 mM Norepinephrine bitartrate/2uM reboxetine/10uM atomoxetine) and incubated at room temperature for 1 hour to allow the binding of ligands to NET. For the apo form, the membrane pellet was used directly for downstream purification.

The membrane was solubilized in a Buffer (25 mM HEPES pH 7.4, 300 mM NaCl, 1% (w/v) DDM (Anatrace), and 0.1% (w/v) CHS (Anatrace)), supplemented with protease inhibitor cocktail and with/without the corresponding ligands, for 1.5 hours at 4°C. The insoluble materials were removed by ultracentrifugation (43,000 rpm for 1.5 hours at 4°C), and the supernatant was incubated with anti-DYKDDDDK G1 affinity resin (GenScript) for 2.5 hours at 4°C. The beads were washed with a buffer (25 mM HEPES pH 7.4, 300 mM NaCl, 0.03% (w/v) DDM, and 0.003% (w/v) CHS, with/without the corresponding ligands), for 10 column volumes, including an ATP wash step. The protein was then eluted with the same wash buffer supplemented with 0.3 mg/mL 3xFLAG peptide (Biotool). Eluted proteins were further purified by size-exclusion chromatography (SEC) using a Superose 6 Increase 10/300 GL column (Cytiva) in a buffer containing 25 mM HEPES pH 7.5, 300 mM NaCl, 5% glycerol, 0.03% DDM, and 0.003% CHS, with/without the corresponding ligands. Peak fractions were collected and concentrated for subsequent experiments.

In vitro nanobody selection for NET bound with different ligands

The full-length NET bound with reboxetine, and the N-terminal truncated NET bound with NE, were used for nanobody screening respectively. The procedure followed a similar protocol including ribosome display and phage displays that was previously described [2]. The corresponding ligands were included throughout the selection process.

For ribosome display, *in vitro* translation of five homemade nanobody mRNA libraries was conducted using the PUREfrex2.1 kit (GeneFrontier). Briefly, biotinylated NET (80 nM) was added to the diluted reaction mixture to start solution panning. Nanobodies bound to their corresponding mRNAs were captured by pulling down biotinylated NET. Following elution, the mRNAs were then purified, reverse transcribed, and amplified by PCR. The PCR products were

then cloned into a modified phage display vector and transformed into SS320 cells via electroporation. Using M13KO7 helper phage (NEB), an initial phage library was produced.

The first round of phage display was conducted in a 96-well format. Biotinylated NET (50 nM) was incubated with purified phages on ice for 20 minutes. The panning solution was then added to pre-blocked NeutrAvidin-coated 96-well plates (NuncMaxisorp, Merck) and incubated for 15 minutes. The wells were washed, and phages were eluted using mild trypsin digestion. Eluted phages were used to infect SS320 cells to amplify phages for a second round of display. In the second round, biotinylated NET (80 nM) was incubated with purified phages in panning solution on ice for 20 minutes. Bound phages were captured by the Dynabeads MyOne Streptavidin C1 (Invitrogen). After washing, weakly associated phages were removed by incubation with 2 μM of non-biotinylated NET on ice. Eluted phages were then used for amplifying phages for a third round of display. The third round was performed similarly to the second round, except that a pre-depletion step using empty C1 beads was included to remove streptavidin-binding nanobodies. Eluted phages from the third round were used to infect SS320 cells for phagemid purification. The final phagemid was extracted using the QIAprep Spin Miniprep Kit (Qiagen). DNA fragments encoding enriched nanobodies were cloned into the expression vector pET26b with an N-terminal pelB signal sequence and a C-terminal His-tag. Positive clones were expressed and identified by ELISA followed by sequencing.

Expression and purification of nanobodies

Desirable clones were transformed into *E. coli* BL21 competent cells, and nanobody overexpression was induced by adding 1 mM isopropyl-β-d-thiogalactoside (IPTG) (goldbio). The cells were cultured for another 16 hours at 22°C in TB medium (BD). Nanobodies were extracted from the periplasmic space using an osmotic shock method [3]. The extract was then cleared by centrifugation at 8,000g for 20 minutes. The supernatant was incubated with 200 μL

of Ni-NTA Superflow (Smart-Lifesciences) for 1 hour at 4°C and then the beads were washed with a buffer (25 mM HEPES pH 7.4, 300 mM NaCl, 25 mM imidazole). Nanobodies were eluted using Washing Buffer supplemented with 400 mM imidazole. The eluted proteins were further purified on a Superdex 75 Increase 10/300 GL (Cytiva) column in buffer containing 25 mM HEPES pH 7.4, 150 mM NaCl, and 5% glycerol.

Nanobody Characterization

A pull-down assay was used to characterize the binding of nanobodies with NET bound with different ligands. His-tagged nanobodies were used as bait, and NET served as prey. Briefly, 2 μg of NET bound with different ligands were incubated with 1 μg of purified nanobody in a 50 μL of reaction buffer (25 mM HEPES pH 7.5, 150 mM NaCl, 0.03% DDM and 0.003% CHS, with/without the corresponding ligands) on ice for 30 minutes. Subsequently, the complex was pulled down using 10 μL of pre-washed Ni-NTA Superflow (Smart-Lifesciences), followed by washing and elution steps. The eluted proteins were analyzed by SDS-PAGE.

Assembly of hNETcryo/nanobody complex

Purified hNETcryo bound with different ligands was incubated with selected nanobodies at a molar ratio of 1:1.5 on ice for 1-3 hours. The complex was then purified on a Superdex 200 Increase 3.2/300 (Cytiva) column in a buffer containing 25 mM HEPES pH 7.4, 150 mM NaCl, 0.03% DDM and 0.003% CHS, with/without the corresponding ligands. Peak fractions were pooled and concentrated for cryo-EM analysis.

Cryo-EM Sample Preparation and Data Collection

A total of 3 μL of the purified hNETcryo in complex with different nanobodies and ligands (~5mg/mL) was applied to glow-discharged (Coolglow, SuPro Instruments) holey carbon

grids (Quantifoil R1.2/1.3 400 mesh). The grids were blotted for 4 seconds at ~100 % humidity and 4 °C, and then plunge-frozen in liquid ethane using the Vitrobot Mark IV (Thermo Fisher Scientific).

All cryo-EM data were collected on a Titan Krios G4 (FEI) operating at 300 kV, equipped with a Selectris X imaging filter (Thermo Fisher Scientific) and a Falcon 4i direct electron detector (Thermo Fisher Scientific). An imaging filter with a slit width of 10 eV was used. All cryo-EM movies were recorded using EPU (Thermo Fisher Scientific), with a total electron dose at ~50 electrons/Å^2. The nominal magnification of 130 kx corresponds to a calibrated pixel size of 0.92 Å on the specimen. The defocus range for the samples was between 1.0 and 2.2 µm.

Image processing

The image processing workflows are illustrated in detail in Fig. S2 and were performed using cryoSPARC v4 [4]. Briefly, motion-correction and dose weighting were performed using the Patch_Motion_Correction module in cryoSPARC v4. After CTF was estimated with the Patch_CTF module, micrographs with CTF-estimated resolution worse than 3.8 Å, high drift values, and extreme defocus range were excluded from further data analysis.

Data analysis was conducted in an iterative manner. Initial particles from ~200 micrographs were picked using topaz [5]. Two-dimensional (2D) classifications were performed on these picked particles, and 2D averages showing nice protein features were used as initial templates for particle picking across all selected micrographs using the Template_Picker module. After 2 rounds of 2D classifications to remove junk particles and false picks, particles belonging to the nice 2D class averages were selected for an Ab-initio 3D reconstruction. Clear secondary structure features were readily visible in the initial 3D reconstruction. This initial 3D volume was then utilized to generate 40 2D templates using the Create_Templates module. These generated

2D templates were served as templates for particle picking across all selected micrographs once again. After 2 rounds of 2D classifications, particles of good quality were subjected to one round of heterogeneous refinement using one good 3D volume and 3 bad 3D volumes. Non-Uniform refinement was then performed on the resulting good class, followed by Local_CTF_refinement and reference_based_motion_correction. The CTF-refined and polished particles were subjected to another round of heterogeneous refinement using one good 3D volume and 3 bad 3D volumes. Particles belonging to the good class were used for the final round of Non-Uniform refinement.

Local resolution variations were estimated in cryoSPARC. Resolutions of the refinement were estimated according to the gold-standard Fourier Shell Correlation (FSC) 0.143 criterion.

Model building and refinement

AlphaFold [6] predictions of the human NET (AF-P23975-F1_v4) and nanobodies were used as initial models for model rebuilding into the cryo-EM density map of high resolution. The initial models were first docked into the cryo-EM density map using UCSF Chimera [7]. Iterative manual rebuilding and adjustment were performed in Coot [8], including with addition of water molecules, ligands, and lipids. Then, the models were refined using Phenix [9]. Structural figures were prepared using USCF Chimera, ChimeraX [10], and Pymol (https://pymol.org/2/).

Cell-based Uptake Assay

Different hNET constructs were transiently transfected into HEK293T cells for the uptake assay. 24 hours after transfection, cells were digested and re-plated at about 150000 cells per well on a poly-L-lysine-coated (0.1mg/mL) 48-well tissue culture plate one day before experiments. Untransfected HEK293T cells were used as a control and treated in the same way. The expression level of hNET constructs was analyzed by western blot using anti-hNET antibody (Abcam, ab254361). On the day of the experiment, cells were counted and washed once with uptake buffer (130mM NaCl, 2mM KCl, 1mM CaCl2, 1mM MgSO4, 10mM HEPES pH

7.4, 5mM D-glucose) at room temperature. The [3H]NE uptake assay was initiated by adding increasing concentrations (100nM to 10uM) of NE mixture ([3H]NE: unlabeled NE at 1:40) diluted in the uptake buffer and the reaction was carried out at room temperature for 20mins. Determined by preliminary experiments, the duration of the assay was set to ensure that the reaction was in the linear range. For determination of IC50 values of inhibitors, washed cells were pre-incubated with increasing concentrations of inhibitors for 10mins at room temperature before adding 100nM of NE mixture ([3H]NE: unlabeled NE at 1:4) for an additional 20mins of uptake reaction at room temperature.

The reactions were stopped by washing cells three times with the ice-cold uptake buffer containing 5uM desipramine. The cells were then lysed in 100 µL of uptake buffer containing 1% Triton X-100 and the total lysate was then transferred to a tube containing 0.6mL scintillating agent (PerkinElmer). Radioactivity was measured by MicroBeta2 Microplate Counter (PerkinElmer). Data was analyzed in GraphPad Prism9 to obtain the Km, Vmax, and IC50 values.

Proteoliposome-based Uptake Assay

Purified hNETcryo was reconstituted into proteoliposome for the proteoliposome-based uptake assay. Purified hNETcryo was mixed with extruded (400nm) liposome (DOPC: DOPE: DOPS: cholesterol at a weight ratio of 54:30:8:8) at a protein-to-lipid molar ratio of 1:4000 in the presence of DDM. The buffer used for reconstitution was 25mM HEPES pH 7.4, 80mM KCl, and 20mM NaCl. Reconstitution was initiated by adding Bio-Beads SM-2 (Bio-Rad) for overnight incubation at 4 °C. For samples containing nanobodies, 2uM of nanobodies were added into the reaction 1 hour after adding Bio-Beads. Reconstituted liposomes were further purified by gradient-centrifugation and concentrated by ultracentrifugation to the same lipid

concentration. To prevent the dissociation of nanobodies, 2uM of nanobodies were included in the buffer throughout the reconstitution.

For initiating the transport assay, reconstituted proteoliposomes were diluted 40 x fold into the transport buffer (25mM HEPES pH 7.4, 150mM NaCl) supplemented with 800nM of NE mixture ([3H]NE: unlabeled NE at 1:9) at room temperature for 30s (pre-determined by a time-course assay to ensure the reaction is in the linear range). Empty liposomes were used as background control and treated in the same way. At the end of the reaction, 5uM desipramine was added to arrest the reaction and then proteoliposomes were captured by a 0.22-μm filter and washed four times (each time with 0.5mL) by filtration method using ice-cold transport buffer supplemented with 5uM desipramine. The filter was then soaked directly into a scintillating agent (PerkinElmer) overnight before radioactivity measurement by MicroBeta2 Microplate Counter (PerkinElmer).

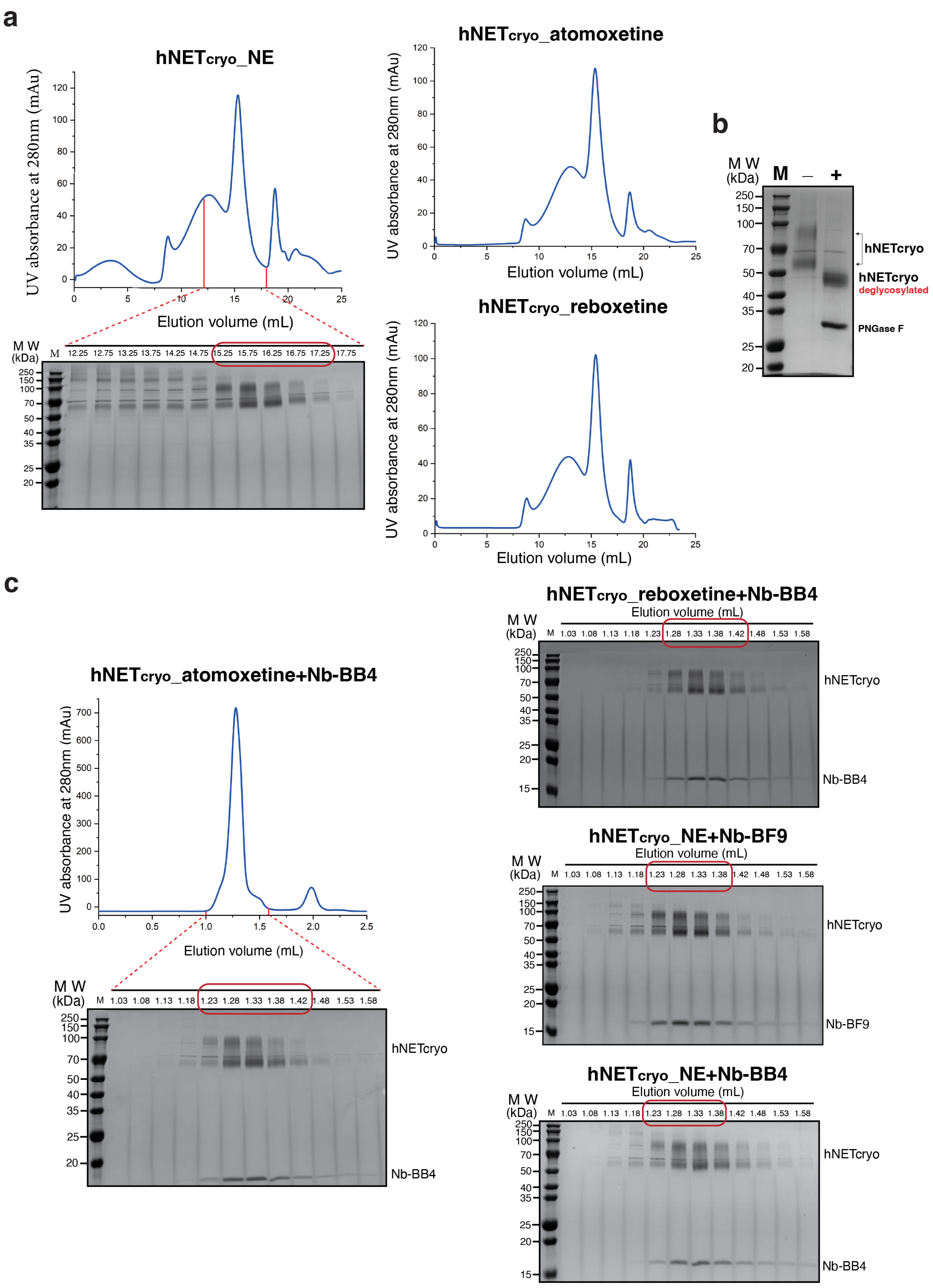

a
hNETcryo_NE
UV absorbance at 280nm (mAu)
Elution volume (mL)
M W (kDa)
M 12.25 12.75 13.25 13.75 14.25 14.75 15.25 15.75 16.25 16.75 17.25 17.75
hNETcryo_atomoxetine
hNETcryo_reboxetine
b
M W (kDa)
M – +
hNETcryo
hNETcryo
deglycosylated
PNGase F
c
hNETcryo_atomoxetine+Nb-BB4
M 1.03 1.08 1.13 1.18 1.23 1.28 1.33 1.38 1.42 1.48 1.53 1.58
hNETcryo
Nb-BB4
hNETcryo_reboxetine+Nb-BB4
hNETcryo_NE+Nb-BF9
Nb-BF9
hNETcryo_NE+Nb-BB4

Figure. S1 | Protein Samples for cryo-EM analysis

a. hNETcryo purified with corresponding ligands was subjected to size-exclusion chromatography. The top left panel illustrates the elution profile of hNETcryo bound with NE, while the bottom left panel shows the analysis of fractions using SDS-PAGE and Coomassie-blue staining. The red circle indicates the fractions pooled for further experiments. The top right panel shows the elution profile of hNETcryo bound with atomoxetine, and the bottom right panel presents the elution profile of hNETcryo bound with reboxetine. **b.** 2 μg of purified hNETcryo bound with NE was treated with 1 μL of PNGase F (NEB) overnight at 4°C. Sample was then analyzed using SDS-PAGE and Coomassie-blue staining. **c.** hNETcryo purified with corresponding ligands was incubated with the indicated nanobody and then subjected to size-exclusion chromatography. The top left panel shows the elution profile of the hNETcryo_atomoxetine_Nb-BB4 complex, while the bottom left panel illustrates the analysis of fractions by SDS-PAGE and Coomassie-blue staining. The red circle indicates the fractions pooled for cryo-EM analysis. The right panel shows the SDS-PAGE gel of fractions from the elution profile of hNETcryo_reboxetine_Nb-BB4, hNETcryo_NE_Nb-BF9, and hNETcryo_NE_Nb-BB4 complexes, respectively.

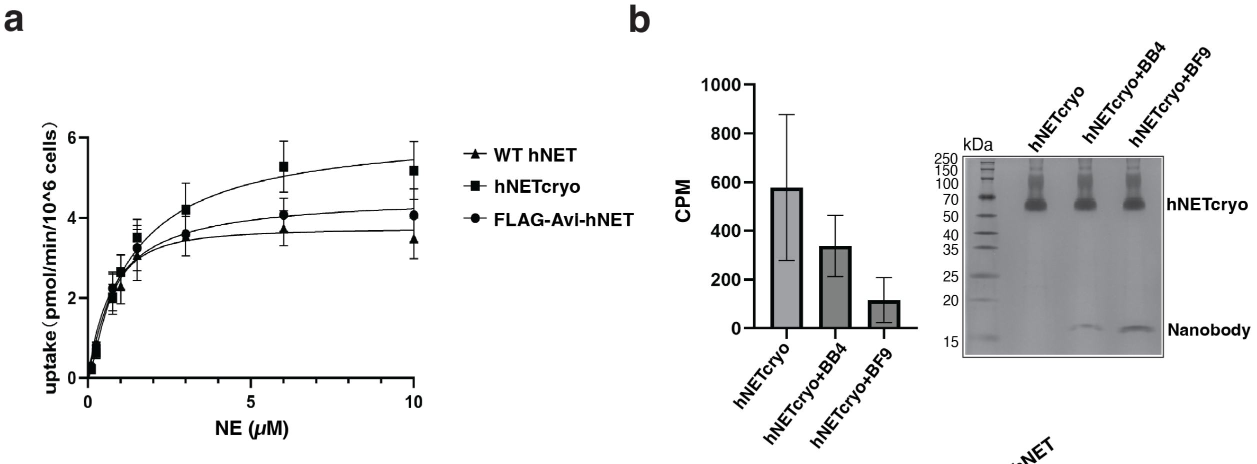

	Km (μM)	Vmax (pmol/min/10^6 cells)
WT hNET	0.74±0.15	4.12±0.23
hNETcryo	1.39±0.22	6.21±0.33
FLAG-Avi-hNET	0.79±0.14	4.56±0.22

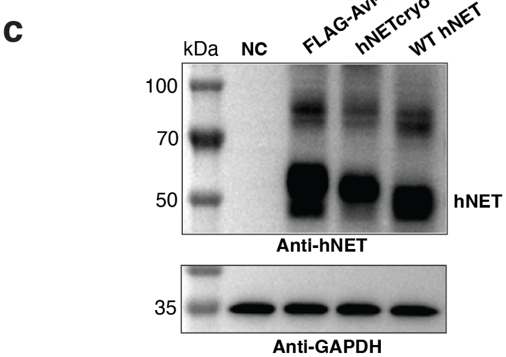

Figure. S2 | Transport Assays of hNET

a. A cell-based uptake assay was used to measure the kinetic parameters of [^{3}H]NE uptake of various NET constructs. The presented data are background subtracted and the means from three independent experiments ± S.E.M., and were fitted using the generalized Michaelis–Menton formulation with the non-linear fitting method. The Km and Vmax values for different constructs are summarized in the table below. WT stands for wild type. **b**. The left panel shows the proteoliposome-based transport assay of [^{3}H]NE in the absence and presence of two nanobodies. The presented data are background subtracted and the means from four independent experiments ± S.E.M. The right panel shows that the amount of hNETcryo used in the assay is consistent. Proteins were analyzed by SDS-PAGE and silver-staining. **c.** The expression levels of hNET constructs in HEK293T cells for **(a)** were analyzed by western blot.

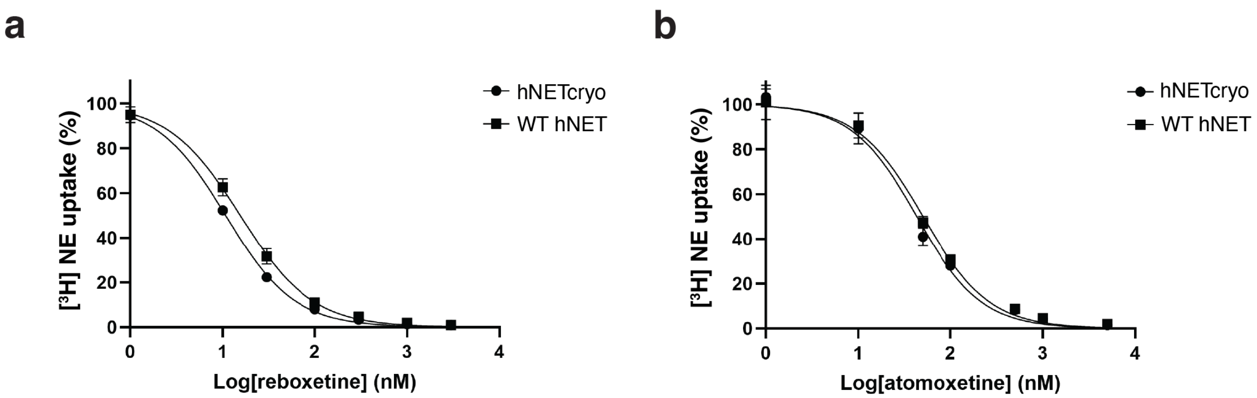

Figure. S3 | Inhibition of NE Transport by Different Inhibitors

a, b. Uptake of [3H]NE by hNETcryo and WT hNET expressed in HEK293T cells. Inhibition of uptake was done by increasing concentrations of reboxetine and atomoxetine, respectively. The data was normalized to the values of zero inhibitor concentration. The presented data are the means from three-independent experiments ± S.E.M. and were fitted to a four-parameter logistic equation, yielding IC50 values 10.51±0.34 nM for hNETcryo and 15.69±0.84 nM for WT hNET for reboxetine, 35.88±2.88 nM for hNETcryo and 44.85±3.41 nM for WT hNET for atomoxetine. WT stands for wild type.

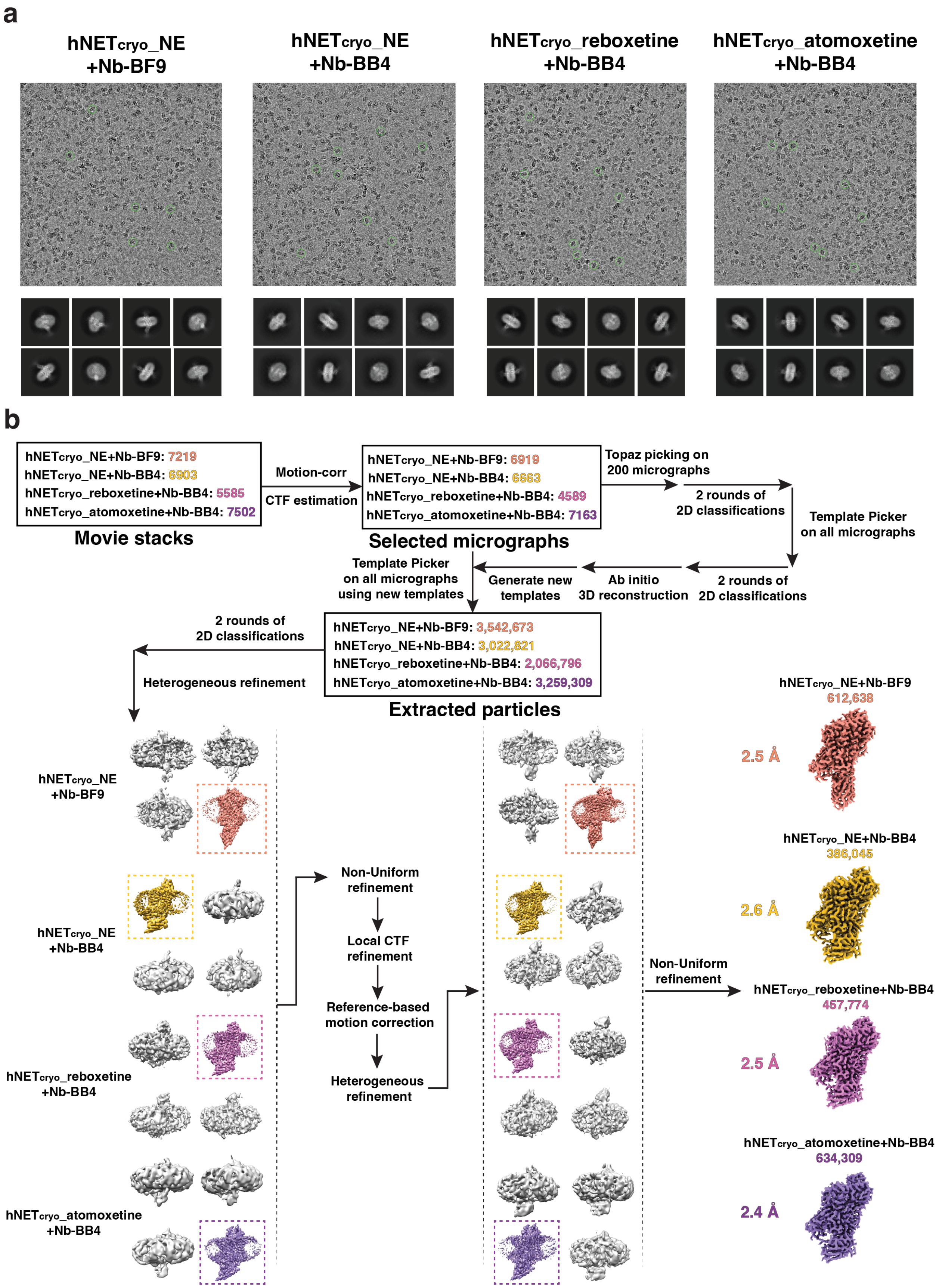
a
hNETcryo_NE
+Nb-BF9
hNETcryo_NE
+Nb-BB4
hNETcryo_reboxetine
+Nb-BB4
hNETcryo_atomoxetine
+Nb-BB4
b
hNETcryo_NE+Nb-BF9: 7219
hNETcryo_NE+Nb-BB4: 6903
hNETcryo_reboxetine+Nb-BB4: 5585
hNETcryo_atomoxetine+Nb-BB4: 7502
Movie stacks
Motion-corr
CTF estimation
hNETcryo_NE+Nb-BF9: 6919
hNETcryo_NE+Nb-BB4: 6663
hNETcryo_reboxetine+Nb-BB4: 4589
hNETcryo_atomoxetine+Nb-BB4: 7163
Selected micrographs
Topaz picking on
200 micrographs
2 rounds of
2D classifications
Template Picker
on all micrographs
2 rounds of
2D classifications
Ab initio
3D reconstruction
Generate new
templates
Template Picker
on all micrographs
using new templates
hNETcryo_NE+Nb-BF9: 3,542,673
hNETcryo_NE+Nb-BB4: 3,022,821
hNETcryo_reboxetine+Nb-BB4: 2,066,796
hNETcryo_atomoxetine+Nb-BB4: 3,259,309
Extracted particles
2 rounds of
2D classifications
Heterogeneous refinement
hNETcryo_NE
+Nb-BF9
hNETcryo_NE
+Nb-BB4
hNETcryo_reboxetine
+Nb-BB4
hNETcryo_atomoxetine
+Nb-BB4
Non-Uniform
refinement
Local CTF
refinement
Reference-based
motion correction
Heterogeneous
refinement
Non-Uniform
refinement
hNETcryo_NE+Nb-BF9
612,638
2.5 Å
hNETcryo_NE+Nb-BB4
386,045
2.6 Å
hNETcryo_reboxetine+Nb-BB4
457,774
2.5 Å
hNETcryo_atomoxetine+Nb-BB4
634,309
2.4 Å

Figure. S4 | Cryo-EM analysis of NET in complex with nanobody, NE, and inhibitors

a. The top images show representative cryo-EM micrographs (defocus at around 2 μm) of the indicated complexes. The green circles, with a diameter of 150 Å, indicate representative particles picked for downstream analysis. The bottom images display some representative 2D class averages during data analysis. **b.** Image processing workflow for the four datasets. Shown are views of 3D reconstructions parallel to the membrane. The classes highlighted in color and boxed were used in the further analysis.

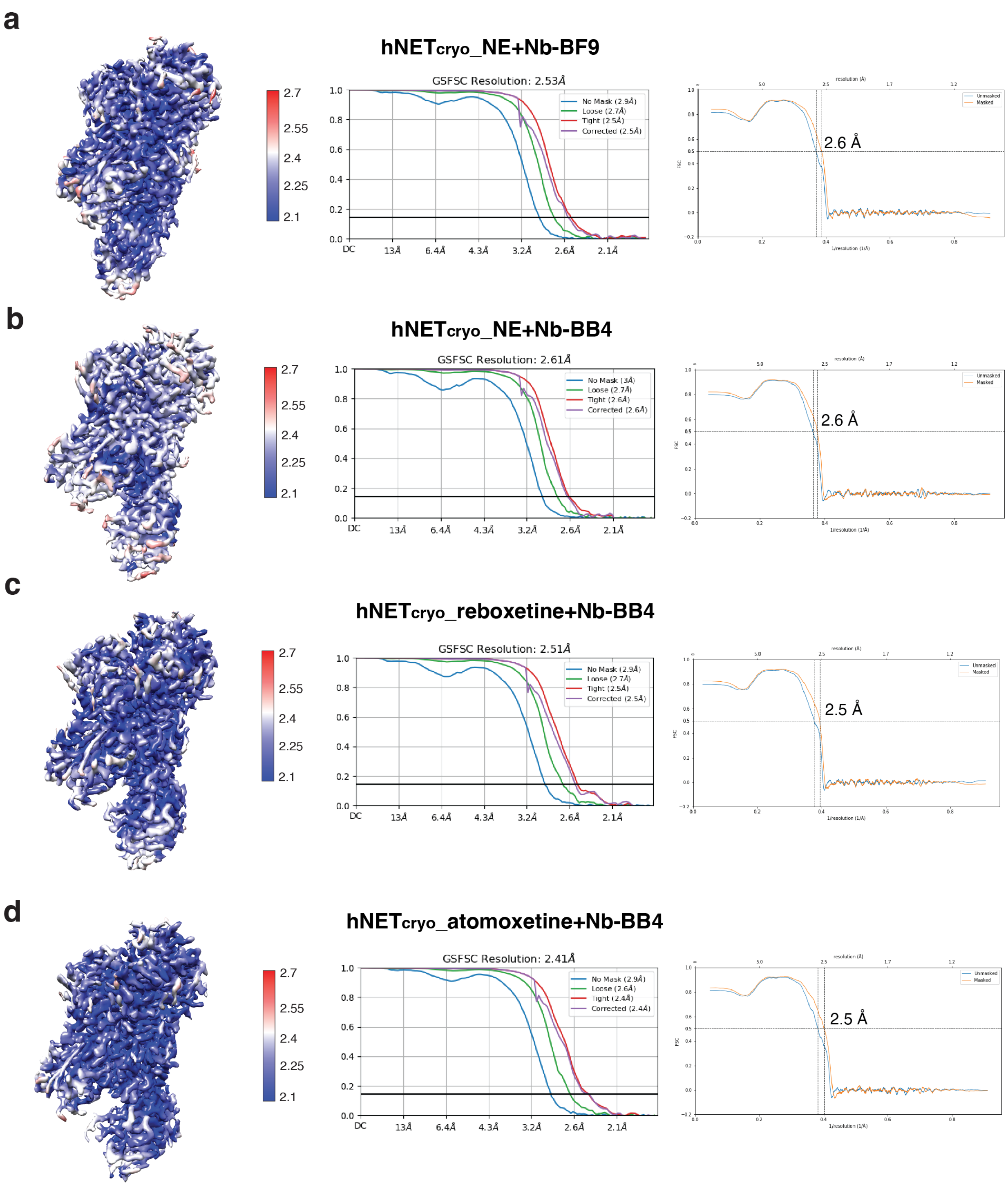

Figure. S5 | The estimated resolution of the cryo-EM reconstructions

a. The left panel shows the local resolution estimation of the cryo-EM map of the hNETcryo_NE+Nb-BF9 complex. The final map was colored based on local resolution, and the scale bar represents values in angstroms (Å). The middle panel is the Golden Standard Fourier

Shell Correlation (GSFSC) curve for the 3D reconstruction. The black line indicates the 0.143 criterion. The right panel shows the FSC curves (masked or unmasked) comparing the refined model of the indicated complex to the summed map used for refinement. The resolution of the model was assessed using the 0.5 criterion of the masked curve. **b, c, d.** The same information as (**a**) is provided for hNETcryo_NE+Nb-BB4, hNETcryo_reboxetine+Nb-BB4, hNETcryo _atomoxetine+Nb-BB4, respectively.

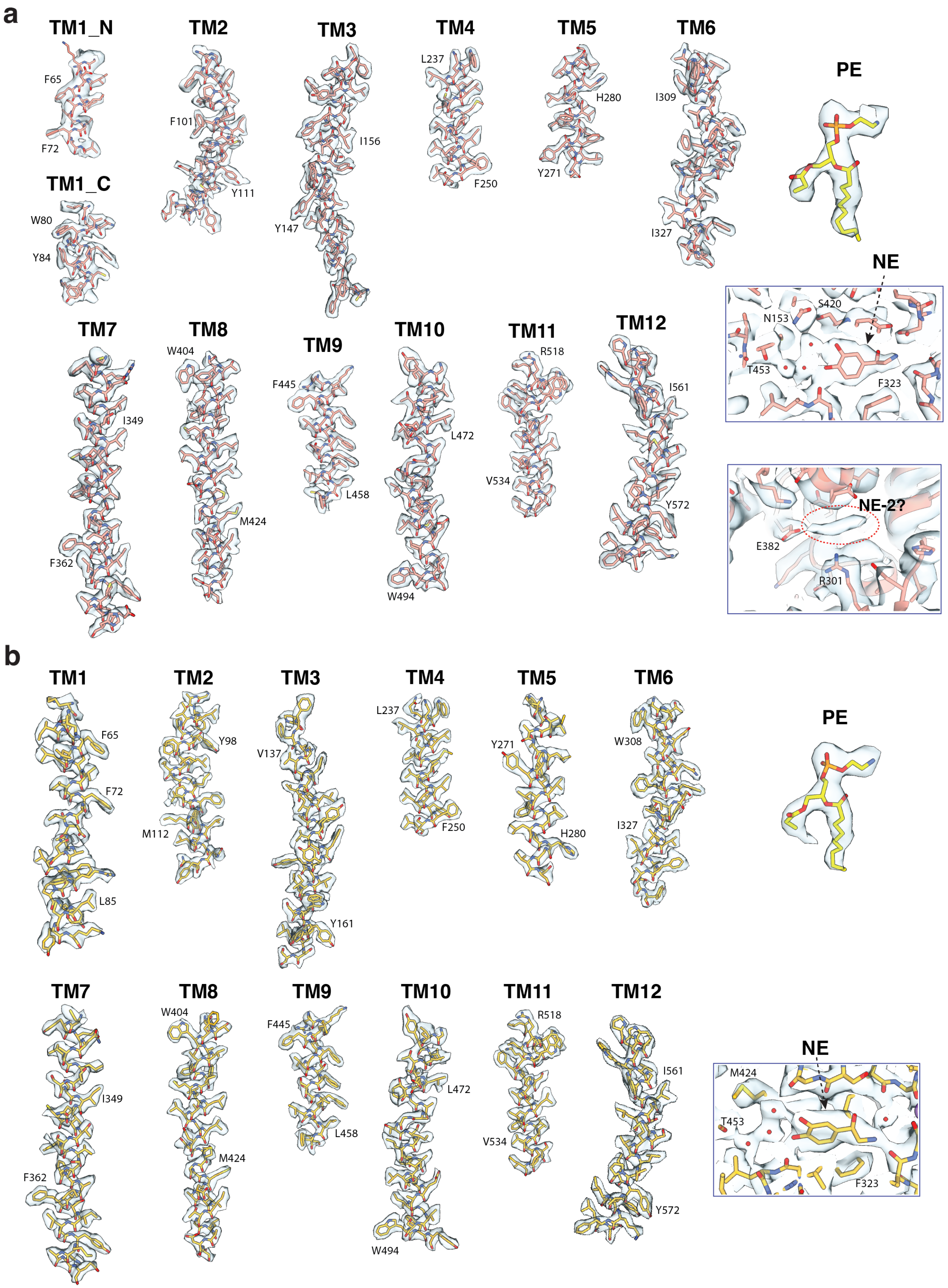
a
TM1_N
F65
F72
TM1_C
W80
Y84
TM2
F101
Y111
TM3
I156
Y147
TM4
L237
F250
TM5
H280
Y271
TM6
I309
I327
PE
NE
S420
N153
T453
F323
TM7
I349
F362
TM8
W404
M424
TM9
F445
L458
TM10
L472
W494
TM11
R518
V534
TM12
I561
Y572
NE-2?
E382
R301
b
TM1
F65
F72
L85
TM2
Y98
M112
TM3
V137
Y161
TM4
L237
F250
TM5
Y271
H280
TM6
W308
I327
PE
TM7
I349
F362
TM8
W404
M424
TM9
F445
L458
TM10
L472
W494
TM11
R518
V534
TM12
I561
Y572
NE
M424
T453
F323

Figure. S6 | Cryo-EM densities and fitted models for representative regions of NET in complex with NE

a. Cryo-EM densities with fitted model for NET_NE_I. The unsharpened map, contoured at 0.035, was used to show the fitting of TM1_N and the putative lipid PE, as well as the location of the potential allosteric binding site for NE-2 (circled with red dashed line). For other parts of the protein, a sharpened map contoured at 0.16, was used. **b.** Densities and model for the NET_NE_Oc. The unsharpened map, contoured at 0.04, was used for showing fitting of the putative lipid PE. The sharpened map was contoured at 0.2.

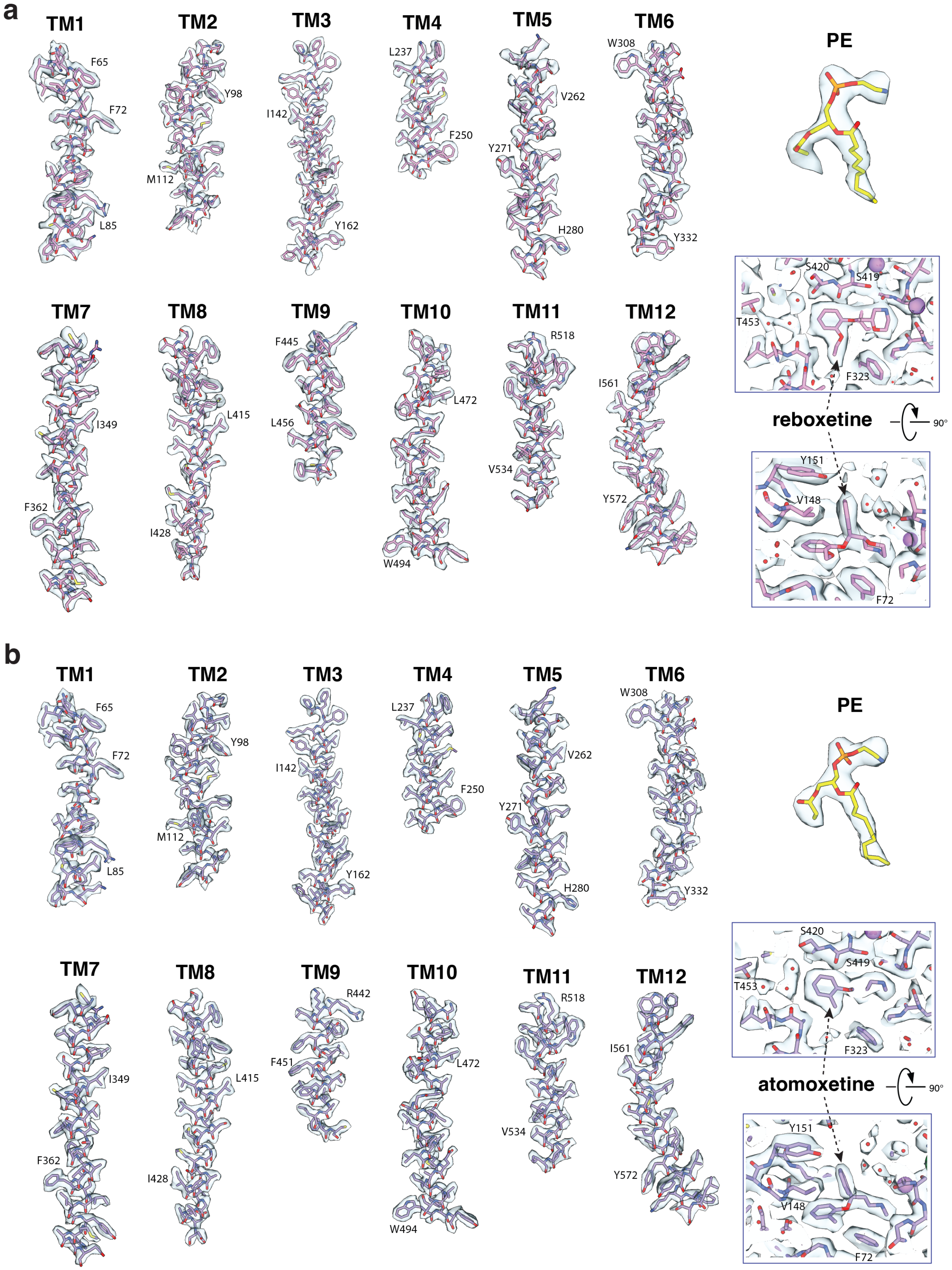
a
TM1
F65
F72
L85
TM2
Y98
M112
TM3
I142
Y162
TM4
L237
F250
TM5
V262
Y271
H280
TM6
W308
Y332
PE
TM7
I349
F362
TM8
L415
I428
TM9
F445
L456
TM10
L472
W494
TM11
R518
V534
TM12
I561
Y572
S420
S419
T453
F323
reboxetine
90°
Y151
V148
F72
b
TM1
F65
F72
L85
TM2
Y98
M112
TM3
I142
Y162
TM4
L237
F250
TM5
V262
Y271
H280
TM6
W308
Y332
PE
TM7
I349
F362
TM8
L415
I428
TM9
R442
F451
TM10
L472
W494
TM11
R518
V534
TM12
I561
Y572
S420
S419
T453
F323
atomoxetine
90°
Y151
V148
F72

Figure. S7 | Cryo-EM densities and fitted models for representative regions of NET in complex with inhibitors

a. Cryo-EM densities with fitted model for the NET_reboxetine_O. The unsharpened map, contoured at 0.04, was used to show fitting of the putative lipid PE. For other parts of the protein, sharpened map contoured at 0.18 was used. **b.** Densities and model for the NET_atomoxetine_O. Maps were contoured at the same level as in (**a**).

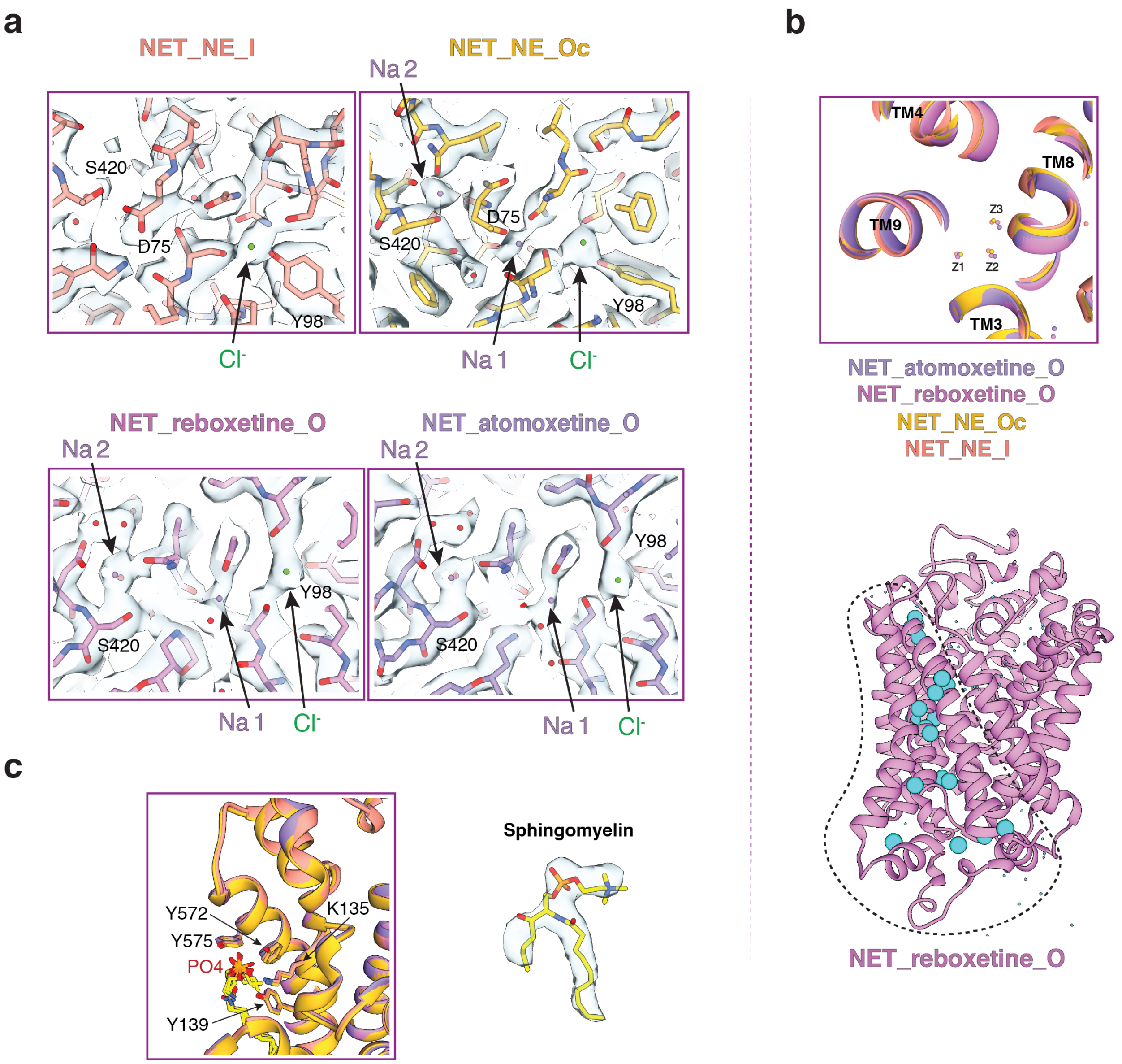

Figure. S8 | Waters, ions, and lipids in different structures

a. Cryo-EM densities with fitted models for all four structures near the ion-binding sites. The contour of the maps is the same as those in Fig. S6 and S7. **b.** The top panel shows the alignment of all four structures, focusing on the water triad region. Three water molecules (Z1, Z2, Z3) are shown in sphere. The bottom panel shows the model of NET_reboxetine_O with water molecules shown as sphere in cyan, and protein shown as cartoon in pink. The big spheres are water molecules with consistent positions across all four structures. The area circled with dashed

line corresponds to protein regions that undergo very minimal movement during conformational changes as shown in (**Fig. 1f, 1g**). **c.** The left panel shows the alignment of all four structures. The putative lipid PE and residues coordinating the phosphate group are shown as sticks. The panel on the right shows a putative lipid sphingomyelin fitted into the density map of NET_atomoxetine_O (unsharpened map, contoured at 0.04).

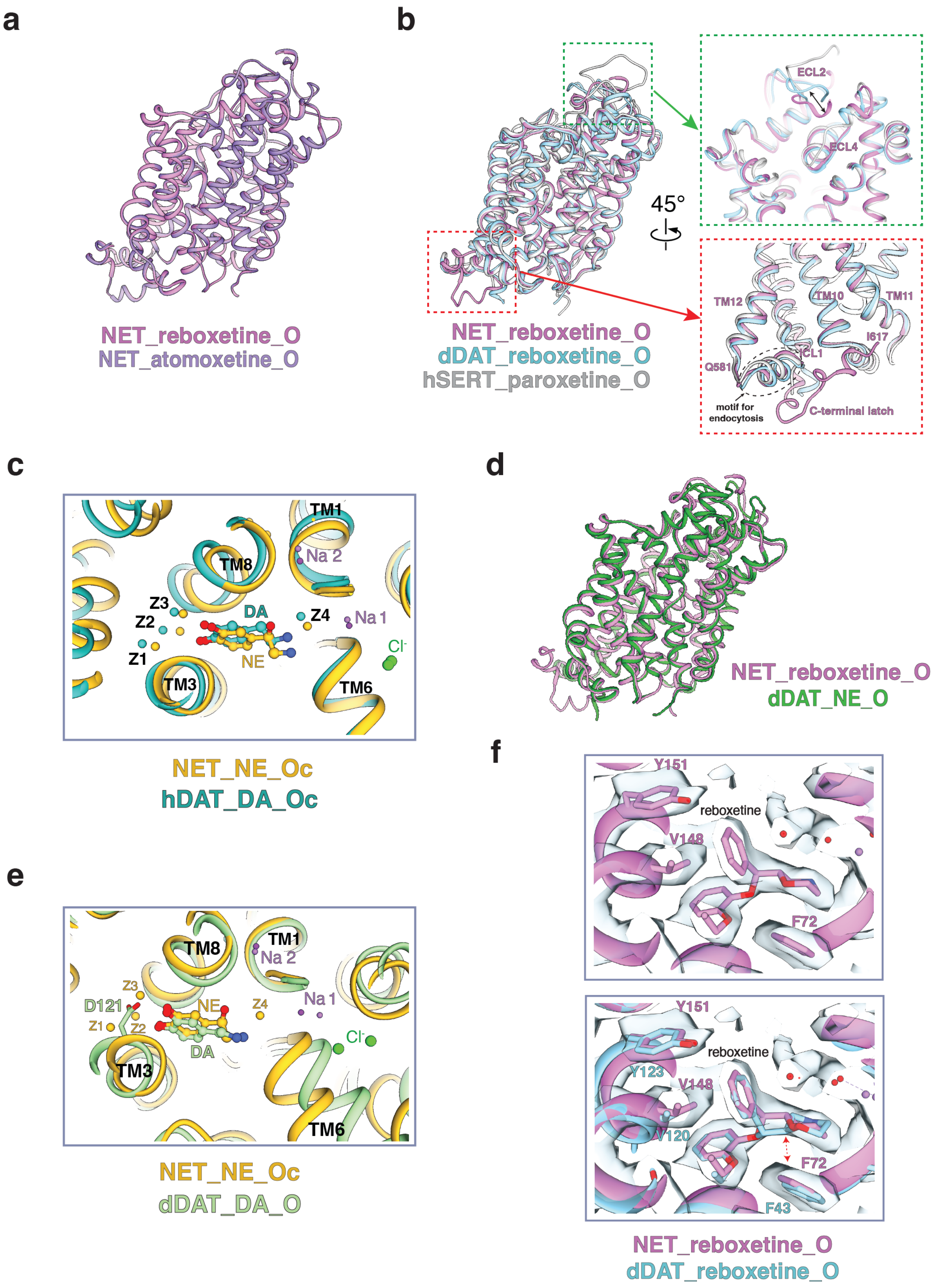
a
NET_reboxetine_O
NET_atomoxetine_O
b
45°
ECL2
ECL4
TM12
TM10
TM11
I617
ICL1
Q581
motif for endocytosis
C-terminal latch
NET_reboxetine_O
dDAT_reboxetine_O
hSERT_paroxetine_O
c
TM1
Na 2
TM8
Z3
Z2
Z1
DA
NE
Z4
Na 1
Cl-
TM3
TM6
NET_NE_Oc
hDAT_DA_Oc
d
NET_reboxetine_O
dDAT_NE_O
e
TM8
TM1
Na 2
Z3
D121
Z1
Z2
NE
Z4
Na 1
DA
Cl-
TM3
TM6
NET_NE_Oc
dDAT_DA_O
f
Y151
reboxetine
V148
F72
Y151
reboxetine
Y123
V148
V120
F72
F43
NET_reboxetine_O
dDAT_reboxetine_O

Figure. S9 | Structural Comparisons of hNET and dDAT

a. Structure alignment of NET_reboxetine_O with NET_atomoxetine_O. **b**. The left panel shows the structure alignment of NET_reboxetine_O with dDAT_ reboxetine_O (PDB: 4XNX) and hSERT_paroxetine_O (PDB: 5I6X). The right panels are the zoomed-in views of the extracellular loop and the C-terminal latch regions of the model. **c.** Structure alignment of NET_NE_Oc with hDAT_DA_Oc (PDB: 8Y2D). Only the substrate-binding site was zoomed in and shown. Water molecules are shown as spheres and colored based on their corresponding models. Na^+ and Cl^- are shown as spheres and colored in purple and green, respectively. **d.** Structure alignment of NET_reboxetine_O with dDAT_NE_O (PDB: 6M0Z). **e.** Structure alignment of NET_NE_Oc with dDAT_DA_O (PDB: 4XP1). Only the substrate-binding site was zoomed in and shown. Water molecules are shown as spheres and colored based on their corresponding models. Na^+ and Cl^- are shown as spheres and colored in purple and green, respectively. **f.** The upper panel shows the model of NET_reboxetine_O fitted in the cryo-EM density map (contoured at 0.018). The lower panel shows the structural alignment of NET_reboxetine_O with dDAT_reboxetine_O (PDB: 4XNX), in the presence of the density map. It should be noted that reboxetine was modeled as the (R,R)-isomer in dDAT_reboxetine_O, but the original study suggested that both isomers could fit. The red dashed line indicates the location of clashes with F72 if the (R,R)-isomer was modeled in NET_reboxetine_O. The reboxetine binding pocket in dDAT is highly conserved but slightly larger than that in hNET, which could thus accommodate reboxetine in more flexible ways. Notable differences are residues F43 (SA) and V120 (SC) of dDAT, likely caused by subtle differences in the overall structure.

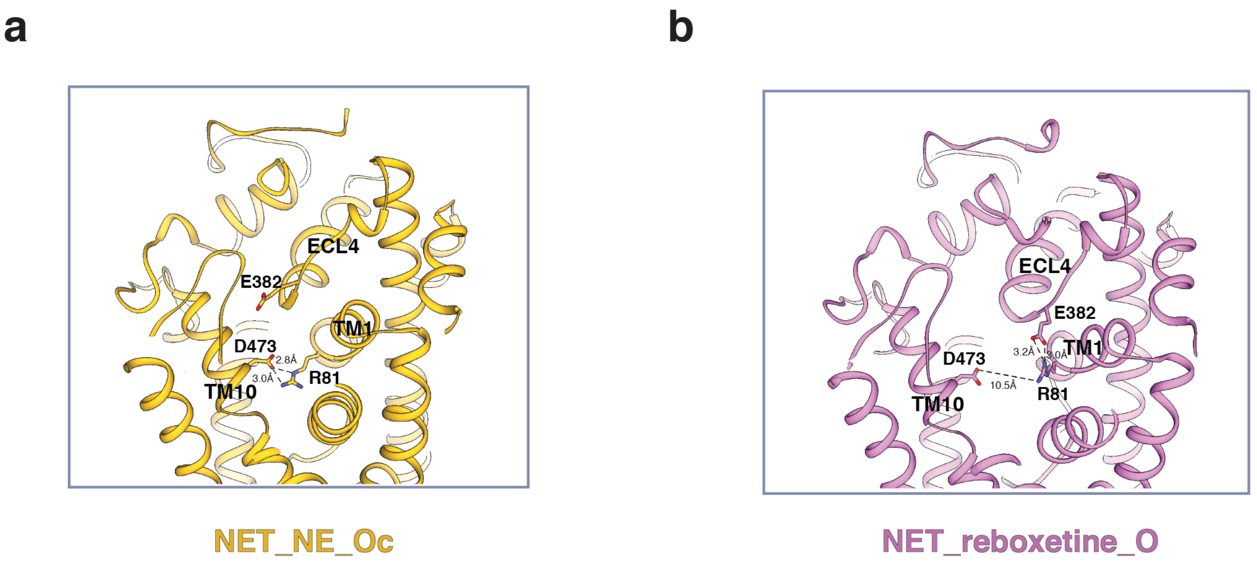

Figure. S10 | Residues Important for Conformational Transition of hNET

a. Model of NET_NE_Oc is shown in cartoon mode. Important residues involved in conformational transition are shown as sticks. The dashed line with number shows the distance between different atoms. **b.** Same as **(a)**, but for the model of NET_reboxetine_O.

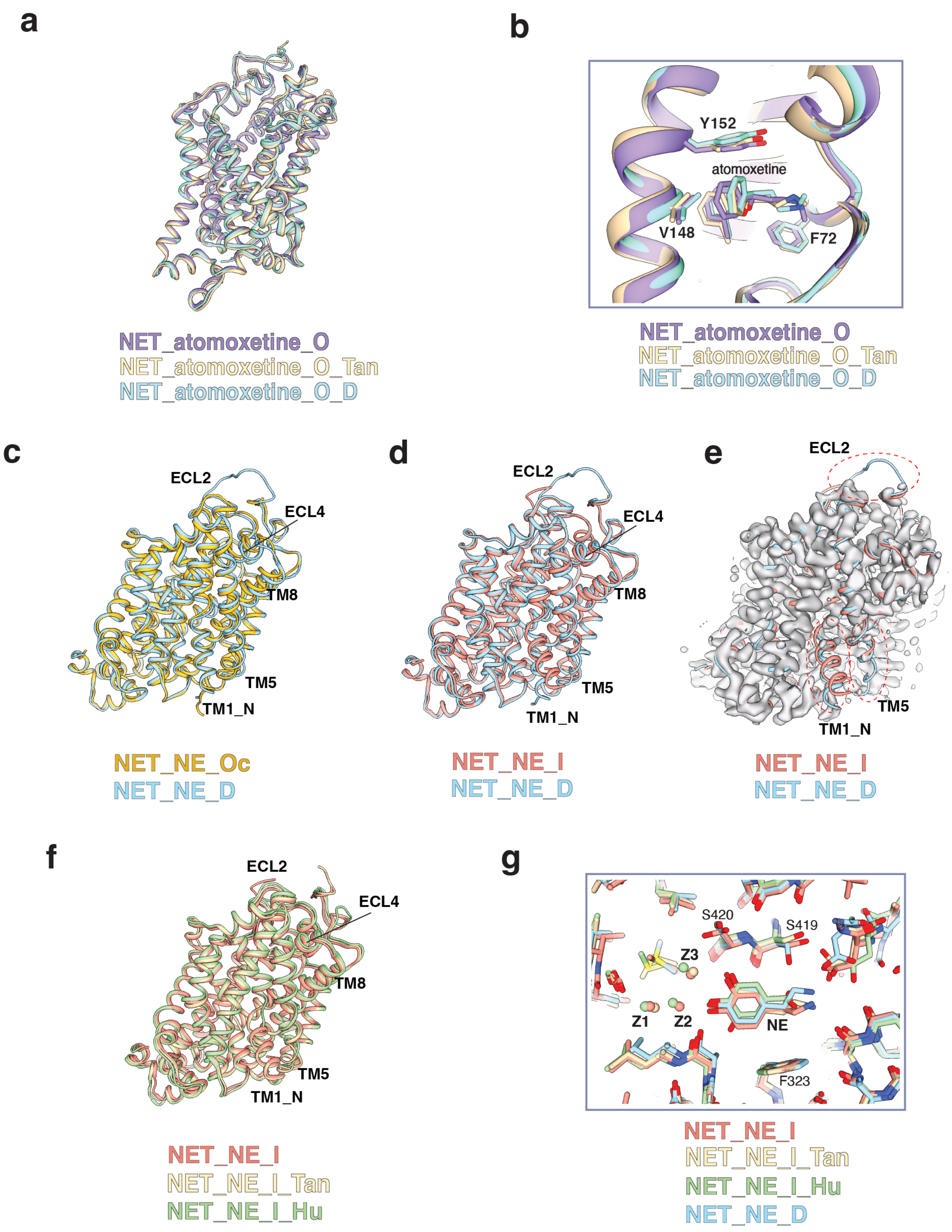
a
NET_atomoxetine_O
NET_atomoxetine_O_Tan
NET_atomoxetine_O_D
b
Y152
atomoxetine
V148
F72
NET_atomoxetine_O
NET_atomoxetine_O_Tan
NET_atomoxetine_O_D
c
ECL2
ECL4
TM8
TM5
TM1_N
NET_NE_Oc
NET_NE_D
d
ECL2
ECL4
TM8
TM5
TM1_N
NET_NE_I
NET_NE_D
e
ECL2
TM5
TM1_N
NET_NE_I
NET_NE_D
f
ECL2
ECL4
TM8
TM5
TM1_N
NET_NE_I
NET_NE_I_Tan
NET_NE_I_Hu
g
S420
S419
Z3
Z1
Z2
NE
F323
NET_NE_I
NET_NE_I_Tan
NET_NE_I_Hu
NET_NE_D

Figure. S11 | Structural Comparisons with reported hNET structures

a. Structure alignment of NET_atomoxetine_O with dimeric hNET bound with atomoxetine (NET_atomoxetine_O_D) (PDB: 8Y92) and monomeric hNET reported by Tan et al. (NET_atomoxetine_O_Tan) (PDB: 8Z1L). **b.** A close-up view of **(a)** on the inhibitor binding site. The ethylamine side chain of atomoxetine was modeled differently so that the interaction between the secondary amine group of atomoxetine and the carbonyl oxygen of F72 is not observed in the other two structures. **c.** Structure alignment of NET_NE_Oc with NET_NE_D (PDB: 8Y95). **d.** Structure alignment of NET_NE_I with NET_NE_D (PDB: 8Y95). It should be noted that the conformation of ECL4 and TM8 is more similar between these two structures. However, TM1_N and the cytosolic side of TM5 were modeled differently. **e.** The same alignment as **(d)** in the presence of cryo-EM density map of the dimer (EMDB-39070, contoured at 0.35, shown in grey). Circles with red dashed line are regions with weak density for model buildings, indicating local flexibility. The weak density could indicate ambiguity in the modeling of TM1_N and the cytosolic side of TM5. **f.** Structure alignment of NET_NE_I with hNET bound with NE reported by Tan et al. (NET_NE_I_Tan) (PDB: 8HFF) and by Hu et al. (NET_NE_I_Hu) (PDB: 8WTV). **g.** Structure alignment of NET_NE_I with NET_NE_I_Tan (PDB: 8HFF), NET_NE_I_Hu (PDB: 8WTV), and NET_NE_D (PDB: 8Y95). A close-up view of the NE binding site is shown. Water molecules (Z1, Z2, Z3) resolved in different structures are shown as spheres, colored accordingly. In NET_NE_I_Tan, two water molecules of the water triad were modeled, while the entire water triad was modeled in NET_NE_I_Hu. On the other hand, no water molecules were resolved in NET_NE_D.

Table S1. Statistics for data collection and refinement

Structure	NET_NE_I	NET_NE_Oc	NET_reboxetine_O	NET_atomoxetine_O
**Data Accession**				
PDB	8ZOY	8ZPB	8ZP1	8ZP2
EMDB	EMD-60322	EMD-60331	EMD-60324	EMD-60325
**Data Collection**				
Microscope	Titan Krios G4			
Detector	Falcon 4i			
Voltage (kV)	300			
Automation software	EPU			
Energy filter	10 eV			
Nominal magnification	130k			
Pixel Size (Å)	0.92			
Electron exposure ($e^-/Å^2$)	50			
Defocus range (μm)	-1.2~-2.2	-1.0~-1.8	-1.2~-2.0	-1.2~-2.2
Movies collected	7219	6903	5585	7502
**Reconstruction**				
Software	cryoSPARC v4			
Micrographs used	6919	6663	4589	7163
Particles extracted	3,542,673	3,022,821	2,066,796	3,259,309
Particles used in refinement	612,638	386,045	457,774	634,309
Symmetry	C1	C1	C1	C1
Overall resolution (Å) FSC=0.143 (masked)	2.5	2.6	2.5	2.4
Map sharpening B-factor ($Å^2$)	-108.6	-103.4	-97.2	-98.5
**Model Refinement**				
Software	AlphaFold2, Coot and Phenix			
Initial model used	From AlphaFold2 (AF-P23975-F1_v4)			
Model Composition				
Protein residues	666	667	671	674
Water	70	54	83	73
Ligands	NE: 1	NE: 1	reboxetine: 1	atomoxetine: 1
Lipids	PE: 1	PE: 1	PE: 1	PE: 1
Ions	Cl^-: 1	Na^+:2, Cl^-: 1	Na^+:2, Cl^-: 1	Na^+:2, Cl^-: 1
B factors ($Å^2$)				
Protein	27.8	29.8	29.6	35.7
Ligand	27.5	29.7	28.0	37.4
Water	19.5	24.3	26.4	32.0
R.M.S. deviations				
Bond length (Å)	0.006	0.004	0.004	0.004
Bond angle (˚)	0.74	0.69	0.68	0.67
Ramachandran statistics (%)				
Outliers	0.0	0.0	0.0	0.0
Allowed	2.0	1.7	1.5	1.8
Favored	98.0	98.3	98.5	98.2
MolProbity score	1.40	1.35	1.36	1.39
All-atom clashscore	6.9	6.0	5.4	5.2
Poor rotamers (%)	1.07	1.08	1.24	1.42
Model resolution (Å) FSC threshold=0.5	2.6	2.6	2.5	2.5

Reference:

1. Goehring, A. *et al.* Screening and large-scale expression of membrane proteins in mammalian cells for structural studies. *Nat Protoc* **9**, 2574-2585 (2014).

2. Zimmermann, I. *et al.* Synthetic single domain antibodies for the conformational trapping of membrane proteins. *Elife* **7** (2018).

3. McMahon, C. *et al.* Yeast surface display platform for rapid discovery of conformationally selective nanobodies. *Nat Struct Mol Biol* **25**, 289-296 (2018).

4. Punjani, A., Rubinstein, J.L., Fleet, D.J. & Brubaker, M.A. cryoSPARC: algorithms for rapid unsupervised cryo-EM structure determination. *Nat Methods* **14**, 290-296 (2017).

5. Bepler, T. *et al.* Positive-unlabeled convolutional neural networks for particle picking in cryo-electron micrographs. *Nat Methods* **16**, 1153-1160 (2019).

6. Jumper, J. *et al.* Highly accurate protein structure prediction with AlphaFold. *Nature* **596**, 583-589 (2021).

7. Pettersen, E.F. *et al.* UCSF Chimera--a visualization system for exploratory research and analysis. *J Comput Chem* **25**, 1605-1612 (2004).

8. Emsley, P. & Cowtan, K. Coot: model-building tools for molecular graphics. *Acta Crystallogr D Biol Crystallogr* **60**, 2126-2132 (2004).

9. Adams, P.D. *et al.* PHENIX: a comprehensive Python-based system for macromolecular structure solution. *Acta Crystallogr D Biol Crystallogr* **66**, 213-221 (2010).

10. Pettersen, E.F. *et al.* UCSF ChimeraX: Structure visualization for researchers, educators, and developers. *Protein Sci* **30**, 70-82 (2021).
